# Comparison of injectable platelet-rich fibrin, titanium platelet-rich fibrin, and 0.8% hyaluronic acid applications versus periodontal dressing alone in wound healing after gingivectomy and gingivoplasty operations: randomized controlled clinical study

**DOI:** 10.1007/s00784-026-06860-5

**Published:** 2026-04-14

**Authors:** Özlem Saraç Atagün, Şeyma Çardakci Bahar, Seval Ceylan Şen, Gülbahar Ustaoğlu, Erkan Özcan, Zeynep Cansu Güzel, Merve Inceöz

**Affiliations:** Department of Periodontology, Gülhane Faculty of Dentistry, University of Health Sciences, Ankara, Turkey

**Keywords:** Gingivectomy, Hyaluronic Acid, I-PRF, T-PRF

## Abstract

**Objectives:**

The primary objective of this study was to evaluate the effects of injectable platelet-rich fibrin (I-PRF), titanium-prepared platelet-rich fibrin (T-PRF), and 0.8% hyaluronic acid (HA) gel compared with a naturally healing control group on gingival wound healing and epithelialization following gingivectomy and gingivoplasty in plaque-induced gingival enlargement. Secondary objectives were to assess clinical periodontal parameters and patient-reported outcomes during a 28-day follow-up period.

**Materials and methods:**

The study included four groups: a control group and three test groups treated with I-PRF, T-PRF, and 0.8% HA gel. All patients underwent gingivectomy and gingivoplasty after the first step of periodontal therapy. Pain and burning were recorded using VAS during the first postoperative week, and analgesic consumption and bleeding were also recorded. Oral health–related quality of life was assessed using OHIP-14. Wound healing was evaluated using Mira-2 Tone staining with digital image analysis, the Landry, Turnbull, and Howley (LTH) wound-healing index, and the H₂O₂ foaming test at days 7, 14, 21, and 28, while clinical periodontal parameters were reassessed at day 28.

**Results:**

A total of 60 systemically healthy patients were included in the study: I-PRF (*n* = 15), T-PRF (*n* = 15), 0.8% HA (*n* = 15), and control (*n* = 15). Significant intergroup differences were observed in several clinical and wound-healing parameters. At day 28, the HA group showed significantly lower gingival inflammation and bleeding compared with the I-PRF, T-PRF, and control groups (*p* < 0.05). All intervention groups demonstrated improved wound healing compared with the control group. On day 7, the control group exhibited a significantly larger non-epithelialized area (169,440 ± 23,583) than the HA group (122,262 ± 18,363), representing a mean reduction difference of 47,178. LTH wound-healing scores were significantly higher in the intervention groups than in the control group across all time points (all *p* ≤ 0.005). Pain, burning, and oral health–related quality of life improved significantly over time in all groups (*p* < 0.001), with the HA and I-PRF groups reporting lower discomfort scores.

**Conclusions:**

I-PRF, T-PRF, and HA enhanced epithelial wound healing and patient comfort compared with control, with HA providing superior reductions in gingival inflammation and bleeding. However, due to the subjective nature of some measures and the lack of histological/molecular analyses, further long-term studies are necessary to confirm these clinical outcomes and understand the underlying biological mechanisms.

**Clinical relevance:**

Adjunctive use of HA, I-PRF, and T-PRF may improve soft-tissue healing and patient comfort after gingivectomy. HA may be particularly practical in routine clinical settings because it does not require blood collection or centrifugation. Therefore, the choice of adjunctive treatment may depend on clinical logistics and cost considerations.

**Supplementary Information:**

The online version contains supplementary material available at 10.1007/s00784-026-06860-5.

## Introduction

Gingival enlargement is usually the result of an inflammatory response to bacterial plaque on tooth surfaces [1]. In addition, many factors such as orthodontic appliances, the use of certain medications, and neoplastic conditions can also cause gingival enlargement [2]. Gingivectomy and gingivoplasty operations contribute to periodontal tissue health and aesthetics by removing excess gingival tissues and providing normal physiological contours [2, 3]. Secondary wound healing is observed at the wound site after gingivectomy and gingivoplasty operations. Inflammation, re-epithelialization, granulation tissue formation, matrix production, and tissue remodeling are the distinct yet overlapping stages of healing [4]. Keratinocytes and the extracellular matrix interact during the crucial and intricate re-epithelialization process, allowing cells to move, multiply, and differentiate to restore tissue structure and function [5]. Epithelialization of the wound surface is completed within 7–14 days, but healing with keratinization occurs within 30 days [3, 4]. Given that these surgical procedures create extensive open wound areas, utilizing the post-gingivectomy wound model provides an ideal clinical environment to test the performance of various biological agents in accelerating tissue repair [3, 6].

Using regenerative biological materials is a strategy aimed at improving periodontal parameters as well as accelerating the biological processes of wound healing [7]. HA, a disaccharide polymer, is the most prevalent glycosaminoglycan produced by connective tissue cells and found in the extracellular matrix [8]. HA is involved in various biological processes, including cell differentiation, embryological development, inflammation, and wound healing [9, 10]. HA is regarded as the perfect biomaterial for pharmacological, medicinal, and cosmetic applications [11]. By exerting anti-inflammatory, anti-edematous, and antibacterial effects, HA facilitates the intricate phases of wound healing and is frequently utilized as a bioactive adjunct in surgical periodontal procedures to accelerate tissue repair and enhance the quality of the healing site [12]. In addition, recent clinical studies have demonstrated that adjunctive use of HA may improve periodontal clinical outcomes, including reductions in probing depth and gains in clinical attachment levels, following both surgical and non-surgical periodontal therapy[13–15].

Platelet-rich fibrin (PRF), an autologous second-generation blood product high in leukocytes and platelets, has been utilized in dentistry for regenerative purposes and to support periodontal wound healing [16–19]. I-PRF was created by using additive-free plastic centrifuge tubes and cutting down on the centrifugation time and speed of PRF [20]. Studies have shown that I-PRF containing more regenerative cells and higher concentrations of growth factors can act as a reservoir of growth factors and lead to the transport of key molecules to the application site to improve and support regeneration [21–24]. According to recent research, I-PRF functions as a dynamic gel with extra growth factor release for up to 10 days and offers a three-dimensional fibrin clot network made up of platelets, leukocytes, type I collagen, osteocalcin, and growth factors [25, 26]. T-PRF is a platelet concentrate whose preparation method is based on the hypothesis that titanium tubes may be more effective than glass tubes in activating platelets [27]. Previous studies have demonstrated that T-PRF can enhance wound healing, increase tissue thickness and vascularization, and improve clinical and biochemical outcomes in various periodontal and oral surgical procedures [27–30].

Although these biological agents are widely used, clinical evidence directly comparing the effectiveness of HA with second-generation platelet concentrates (I-PRF and T-PRF) and a non-treated control group in enhancing the stages of secondary wound healing within a standardized surgical model remains limited. Therefore, the primary objective of this randomized controlled clinical study was to utilize the gingivectomy model to evaluate the performance of I-PRF, T-PRF, and 0.8% HA gel relative to a control group in terms of gingival wound healing and epithelialization. The secondary objectives were to assess their impact on clinical periodontal parameters and patient-reported outcomes.

## Material and methods

### Study design and ethical considerations

The clinical research ethics committee of Health Sciences University Gülhane Educational Research Hospital approved the study (2023/85), and all participants signed an informed consent form. The study was conducted following the Declaration of Helsinki, revised in 2013. The trial was registered at ClinicalTrials.gov (ID: NCT06865092). This study adopted a single-center, single-blinded, randomized controlled trial design and followed the Consolidated Standards of Reporting Trials Statement.

The null hypothesis (H0) of the present study was that the application of I-PRF, T-PRF, or 0.8% HA gel would show no significant difference compared to a natural healing control group regarding gingival wound healing, clinical periodontal parameters, and patient-reported outcomes following gingivectomy/gingivoplasty.

A total of four groups were planned in the study: one control group and three test groups.

**HA Group:** Following gingivectomy with the conventional method, 0.8% HA (Gengigel; Ricerfarma, Milan, Italy) is applied to the wound site and covered with a periodontal dressing.

**I-PRF Group:** Following gingivectomy with the conventional method, I-PRF is applied to the wound site and covered with periodontal dressing.

**T-PRF Group:** Following gingivectomy with the conventional method, T-PRF is applied to the wound site and covered with periodontal dressing.

**Control Group:** Following gingivectomy with the conventional method, the wound site is covered only with periodontal dressing, without additional biomaterials.

### Patient selection and sample size calculation

In this prospective clinical study, individuals who visited the Health Sciences University's Gülhane Faculty of Dentistry Periodontology Clinic from May 2023 to November 2025 with gingival complaints were diagnosed with plaque-induced gingival enlargement due to clinical and radiographic examinations.

The research’s main null hypothesis (H0) was that there would be no statistically significant differences between the study groups (I-PRF, T-PRF, HA, and control) or over the follow-up time points regarding gingival wound healing and clinical periodontal parameters. To test this, similar studies for sample size calculations were examined, and the calculation yielding the highest number according to the statistical methods was considered. The sample size was calculated based on the primary outcome of the study, which was the LTH wound healing index at the 1 st week post-surgery. Following the methodology of a similar study by Bahar et al. [3], where the mean LTH scores at the 1 st week were reported as 3.30 ± 0.56 for the test group and 2.52 ± 0.67 for the control group, a standardized effect size (Cohen’s d) of 1.2632 was determined. Using G*Power (version 3.1.9.2) with a 95% confidence level (α = 0.05) and a theoretical power of 0.80, the minimum sample size was calculated as 11 per group. To account for a potential 30% dropout rate, the final sample size was set at 15 participants per group, totaling 60 participants for the four groups.

Inclusion criteria were systemically healthy patients of both sexes aged 18–65 years presenting with plaque-induced gingival enlargement in the maxillary anterior region. Eligible patients had adequate oral hygiene (plaque index < 1), sufficient attached gingiva, and no clinical attachment loss or radiographic bone loss.

Exclusion criteria included a history of periodontal treatment within the previous 6 months, smoking or alcohol use, and the use of antibiotics, immunosuppressive agents, systemic corticosteroids, chemotherapy, radiotherapy, or medications known to cause gingival enlargement within the previous 6 months. Patients with poor communication skills were also excluded.

Initially, 72 participants were screened for eligibility. Of these, 4 did not meet the inclusion criteria, and 8 were successfully managed with the first step of periodontal therapy; therefore, a total of 60 participants (31 females, 29 males) aged 18–42 years across all groups were included in the study.

### Experimental approach

**Fig. 1:**
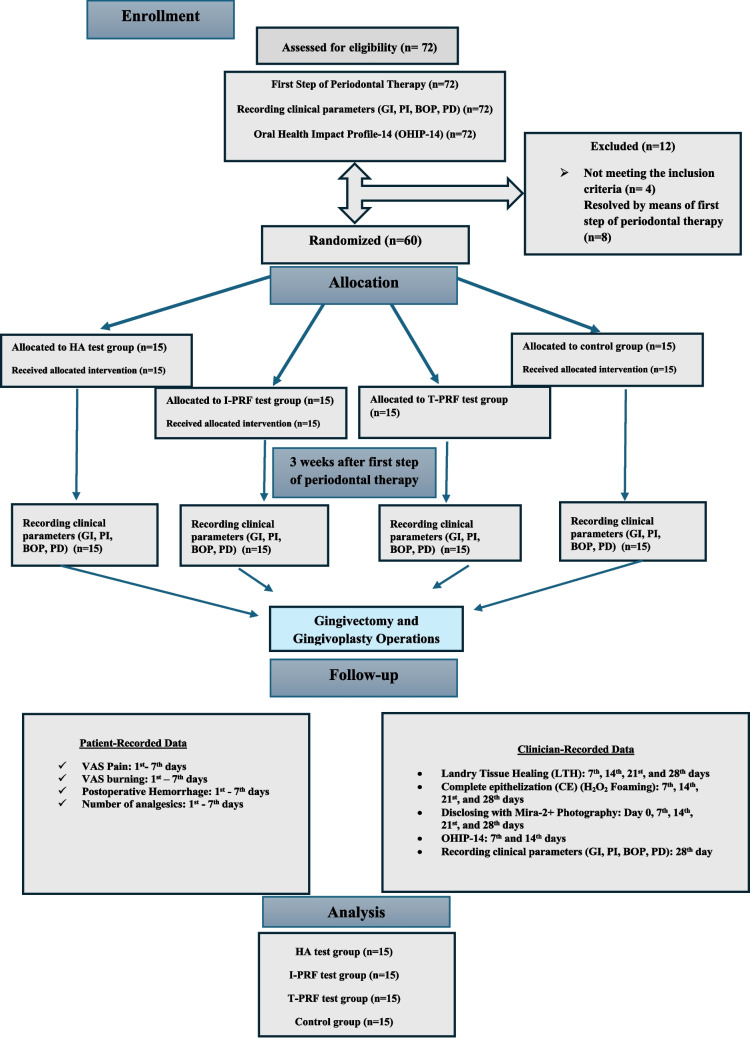
Flowchart of the study

The flowchart is shown in Fig. 1.

All patients diagnosed with plaque-induced gingival enlargement based on clinical and radiographic examinations underwent baseline clinical periodontal assessments followed by the first step of periodontal therapy, including professional mechanical plaque removal (PMPR). Additionally, they received comprehensive oral hygiene instructions. After three weeks, patients were recalled for follow-up, and clinical periodontal parameters were re-evaluated. Gingivectomy and gingivoplasty were performed in patients whose gingival overgrowth remained soft and non-resilient after the first step of periodontal therapy and was limited to six teeth [31].

Gingival growth was graded according to two indices: the buccolingual aspect using the MB index (Seymour [1], later modified by Miranda et al. [32] and the vertical aspect using the GOI index (Angelopoulos and Goaz [33], later modified by Miller et al. [34]. Patients with scores > 0 in both indices were included. Clinical periodontal parameters—including gingival index (GI; Loe and Silness [35]), plaque index (PI; Turesky–Gilmore–Glickman modification of the Quigley–Hein PI [36]), bleeding on probing (BOP [37]), and probing depth (PD [38])—were recorded at six sites per tooth (three buccal: disto-buccal/labial, mesio-buccal/labial, mid-buccal/labial; three lingual: disto-lingual/palatal, mesio-lingual/palatal, mid-lingual/palatal; excluding third molars) using a Williams periodontal probe (122–006, Hu-Friedy). Measurements were performed at baseline (T0), after the first step of periodontal therapy (T1), and at the 1 st month post-gingivectomy and gingivoplasty (T2).

### Randomization and masking

Participants were randomly allocated to the study groups using a block randomization method. The randomization sequence was generated by an independent researcher (S.C.Ş.) using a computer-generated list with fixed block sizes to ensure balanced group distribution. Allocation concealment was achieved using sealed opaque envelopes prepared by the same researcher. The same investigator (O.S.A.) performed the first step of periodontal therapy and all clinical measurements. The investigator responsible for the measurements was blinded to the group assignments throughout the study.

### Examiner calibration

Examiner calibration was performed prior to the study to assess the reliability of clinical measurements. Five patients with plaque-induced gingival enlargement who were not included in the study were examined twice by the same investigator (O.S.A.) with a three-day interval. The second examination was conducted to minimize recall bias and to avoid potential interference from residual plaque-disclosing agents. Intra-examiner reliability was assessed using Cohen’s κ statistics for all clinical parameters evaluated in the study. The obtained κ values were 0.82 for BOP, 0.88 for PI, 0.84 for GI, and 0.86 for PD, indicating substantial agreement.

All subjects' gingivectomy operations were performed by an experienced and calibrated periodontologist (Ş.Ç.B.) who was not involved in the allocation, examination, or statistical analysis. The treatment plan and grouping were also kept confidential for the statistical analyst (G.U.). All participants were unaware of the group assignments during the practice and control sessions and were warned not to mention any details of the operation.

Conventional gingivectomy and gingivoplasty procedures were performed under local infiltration anesthesia (Ultracaine D-S; Sanofi Aventis, Germany). A 45-degree inclined external bevel incision was made using a surgical scalpel (Carbon, No. 15) and a gingivectomy blade (Hu-Friedy 15/16, Chicago, USA), starting from the distal end of the incision line. The interdental area was shaped using an Orban knife (Hu-Friedy 1/2, Chicago, USA), and any remaining granulation tissue was carefully removed from the surrounding area with curettes and scissors (Hu-Friedy, Chicago, USA). Finally, gingivoplasty was completed using a Kirkland knife (Hu-Friedy, Chicago, USA).

After the surgical procedures were completed, the control group areas were left to heal spontaneously. The other groups were treated with I-PRF, T-PRF, or 0.8% HA. Surgical areas in the control and test sites were covered with a zinc oxide-based periodontal dressing that does not contain eugenol (Coepak, Isip, IL, USA). Before applying the dressing, the tooth surfaces were lightly dried to ensure optimal stability. The dressing was then mechanically locked into place in the interproximal spaces. After application, the dressing was adjusted, excess material was removed, and occlusal interferences were carefully checked.

Patients were advised to avoid hot foods, to consume soft foods, and to keep the dressing in the mouth until the next examination. Patients whose periodontal dressing was damaged or dislodged during this period were excluded from the study. Patients were prescribed mouthwash containing 0.12% chlorhexidine and an analgesic containing paracetamol to be used twice a day for one week. Patients were instructed to avoid brushing the wound area until the periodontal dressing was removed after seven days. However, they were advised to maintain oral hygiene in the unaffected areas. Once the periodontal dressing was removed, gentle brushing of the treated area with a soft toothbrush was recommended.

### Preparation of I-PRF

**Fig. 2:**
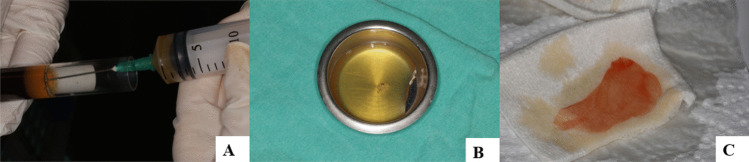
Preparation of the I-PRF

Blood samples were collected in two 10 ml plastic tubes without an anticoagulant. The tubes were centrifuged for 3 min at 2500 rpm in a centrifuge device [39] (Nuve NF 200, Nuve Industrial Materials Manufacturing and Trade Company, Ankara, Turkey). After centrifugation, the I-PRF at the top of the tube was collected through a syringe and transferred to a metal bead. It was left for 15–20 min for polymerization of I-PRF before application to the wound site (Fig. 2).

### Preparation of T-PRF

**Fig. 3:**
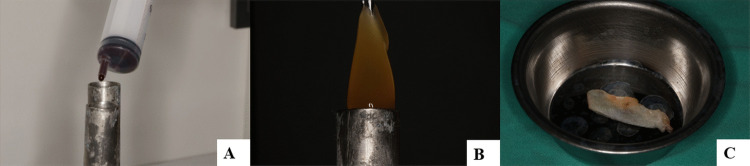
Preparation of the T-PRF

The blood was drawn into a 20 ml syringe and immediately divided into two sterile grade IV titanium tubes containing 10 ml of blood. The tubes were placed opposite each other and centrifuged at 2800 rpm for 12 min at room temperature [40] (Nuve NF 200, Nuve Industrial Materials Manufacturing and Trade Company, Ankara, Turkey). The formed T-PRF clot was carefully extracted from the tube using a sterile dental tweezer, and the underlying red blood cell layer was gently separated. The T-PRF clot was placed between two sterile gauze pads to ensure proper positioning of the buffy coat components on the mesial and distal aspects. Adequate compression was then applied to separate the T-PRF from the serum, forming the membrane (Fig. 3).

### Clinical follow-up of patients

**Fig. 4:**
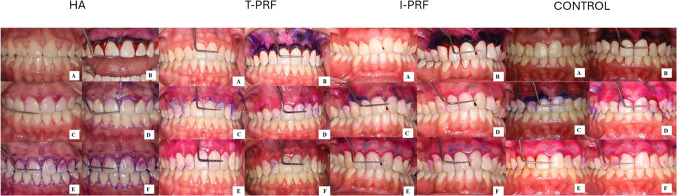
Intraoral photographs

Patients included in the study who underwent gingivectomy and gingivoplasty were recalled for follow-up visits on postoperative days 7, 14, 21, and 28 (Fig. 4).

**Primary outcome measures** of the study were wound epithelialization assessed using Mira-2 Tone solution, the LTH wound healing index, and the H₂O₂ foaming test, which together were used to evaluate soft-tissue healing dynamics.

**Secondary outcome measures** included clinical periodontal parameters (GI, PI, BOP, PD), and patient-reported outcomes such as pain and burning assessed by VAS during the first 7 days, analgesic consumption, postoperative bleeding, and oral health–related quality of life evaluated using the OHIP-14 questionnaire.

### Photographing and evaluating the operation areas

**Fig. 5:**
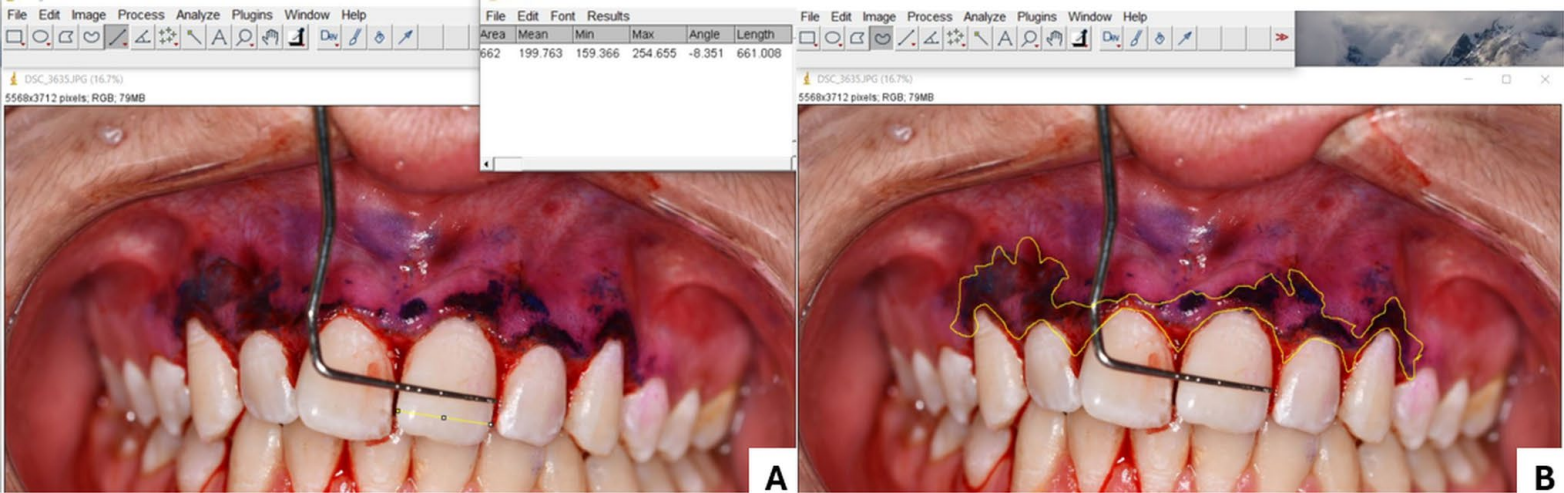
Calculating the stained area in the ImageJ program

On postoperative days 0, 7, 14, 21, and 28, the amount of epithelialization in the wound area was measured using Mira-2 tone solution, a plaque-disclosing agent. The non-epithelialized wound areas during the healing process following gingivectomy were analyzed on standardized digital photographs using ImageJ software (National Institutes of Health, USA; ImageJ version 1.48 V) (Fig. 5). During photography, a Williams periodontal probe was used to scale the shot size and served as a millimetric reference for spatial calibration. To maintain uniformity, every photo was taken by the same individual (M. İ.) using the same camera at the same distance (20 cm), angle, and light level (ISO-800) (Canon EOS R10, Shimomaruko, Ohta-ku, Tokyo, Japan). For each image, the “Set Scale” function in ImageJ was used to convert pixel measurements into real metric units using the known markings of the Williams periodontal probe as a reference, enabling standardized area measurements. Each patient's real intraoral dimensions (mesial to distal) of the maxillary right central incisor were measured and compared to the images' right central incisor dimensions, and the images were calibrated using the ratio of the actual to photographic size to ensure that the measurements were conducted in the same focal plane. To assess intra-examiner reliability, epithelialization measurements were repeated weekly on photographs from ten gingivectomy patients who were not included in the study. [41].

### LTH index

The LTH index was used to measure the healing of wound areas. This index, which ranged from 1 (very poor healing) to 5 (excellent healing), categorized the healing process based on granulation tissue, suppuration, redness, epithelialization, and bleeding [42].

### H2O2 foaming test

Clinical evaluation of wound epithelialization completion was conducted using the H_2_O_2_ foaming test. After using gauze to dry the wound, an injector was used to apply 3% hydrogen peroxide (H_2_O_2_), and the wound was then monitored [43].

### Postoperative pain and burning assessment

The visual analog scale (VAS) is a simple, reliable, and rapidly applied method for measuring the intensity of pain and burning [44, 45]. Post-operative pain and burning were evaluated by VAS. In this method, patients were asked to rate their pain and burning during the first 7 postoperative days as a score between 1 (less pain, less burning) and 10 (severe pain, severe burning).

### Oral health impact profile assessment

OHIP-14 is a 14-item questionnaire designed to assess the impact of various aspects of oral health across seven domains: functional limitations, physical pain, psychological discomfort, physical disability, social disability, and handicap [46]. Higher OHIP-14 scores reflect a more negative self-perception of quality of life, indicating a lower overall oral health-related quality of life. OHIP-14 was completed at baseline and on days 7 and 14 after gingivectomy during a face-to-face interview. Patients answered to the frequency of their negative experiences by following a 5-point Likert-type scale: 0-never, 1-rarely, 2-occasionally, 3-quite often, and 4-very often. The total score ranged from 0 to 56.

### Statistical analysis

Descriptive statistics of the data (frequency, percentage, mean, standard deviation, median, and 25th–75th percentiles) were calculated. Age differences between groups were analyzed using the Mann–Whitney U test, and gender distribution differences were analyzed using the Pearson Chi-Square test. The assumption of normality was assessed using the Shapiro–Wilk test, homogeneity of variances was evaluated with Levene’s test, and the sphericity assumption was examined using Mauchly’s W test. For comparisons of three or more independent groups with normally distributed data, one-way analysis of variance (ANOVA) was applied, whereas the Kruskal–Wallis test was used when the normality assumption was not met. To evaluate differences among three or more related groups with normally distributed data, repeated-measures ANOVA with Greenhouse–Geisser correction was performed; otherwise, the Friedman test was used. Post hoc analyses were conducted using Bonferroni, Tamhane, and adjusted Bonferroni tests to identify the groups responsible for statistically significant differences. Associations between categorical variables were analyzed using Pearson’s chi-square test when the sample size assumption (expected cell count > 5) was met, and Fisher’s exact test when this assumption was violated. For comparisons of dependent categorical variables, Cochran’s Q test was applied, followed by pairwise comparisons using the McNemar test with Holm–Bonferroni correction. All statistical analyses were performed using IBM SPSS Statistics version 27.

## Results

No significant differences were observed between the study groups in terms of age (p = 0.799) or gender distribution (*p* = 0.978) (Supplementary Tables 1–2).

The distributions of clinical measurements and OHIP scores according to study groups and measurement time points are presented in Table 1. Intergroup analyses revealed statistically significant differences among the study groups for day-28 GI (*p* = 0.024), baseline PI (*p* = 0.020), day-28 BOP (*p* < 0.001), and PD measurements at days 21 and 28 (*p* = 0.024 and *p* = 0.001, respectively). Post hoc analyses further showed that the HA group had significantly lower day-28 GI and BOP values than the I-PRF, T-PRF, and control groups (all *p* < 0.05). For PI, a significant baseline difference was observed between the HA and T-PRF groups, with higher values in the HA group (*p* = 0.020). Regarding PD, the control group showed significantly higher values than the T-PRF group at day 21 and then both the HA and T-PRF groups at day 28 (all *p* < 0.05). Time-based analyses demonstrated statistically significant differences in GI and PD measurements across all groups, and in PI and BOP measurements in the HA, I-PRF, and control groups (*p* < 0.05).

**Table 1:** Distributions and comparisons of clinical measurements and OHIP Scores according to study groups and measurement times

		Hyaluronic Acid		I-PRF		T-PRF		Control		Between Groups		
	Between Times	Mean ± SD	M.(25%−75%Q.)	Mean ± SD	M.(25%−75%Q.)	Mean ± SD	M.(25%−75%Q.)	Mean ± SD	M.(25%−75%Q.)	Test Statistic	*p*	Effect Size
***GI***	Baseline	1.46 ± 0.57	1.25(1.12–1.88)	1.21 ± 0.51	1.16(0.75–1.63)	1.32 ± 0.26	1.3(1.13–1.5)	1.31 ± 0.48	1.29(1.13–1.58)	0.936†	0.817	-
	21 st day	0.83 ± 0.48	0.94(0.35–1.17)	1.06 ± 0.59	1.21(0.46–1.52)	1.21 ± 0.29	1.25(1.08–1.46)	1.17 ± 0.54	1.13(1–1.72)	1.884	0.143	-
	28th day	0.49 ± 0.45	0.32(0.07–1.08)	0.9 ± 0.5	1.13(0.29–1.33)	0.97 ± 0.32	0.92(0.79–1.25)	0.99 ± 0.47	1.05(0.63–1.38)	9.434†	0.024*	0.187
	**Test Stat./p**	26.533‡	< 0.001*	7.104	0.003*	6.998	0.003*	6.729	0.004*			
	Effect Size	0.662		0.337		0.333		0.325				
***PI***	Baseline	1.55 ± 0.63	1.29(1.08–2.41)	1.04 ± 0.62	0.83(0.58–1.54)	1.04 ± 0.78	0.77(0.5–1.17)	1.25 ± 0.57	1.08(1–1.33)	9.828 †	0.020*	0.100
	21 st day	0.63 ± 0.37	0.63(0.38–1)	0.82 ± 0.56	0.72(0.41–1.08)	0.72 ± 0.35	0.79(0.58–1.04)	0.89 ± 0.37	0.92(0.58–1.13)	1.069	0.370	-
	28th day	0.79 ± 0.45	0.79(0.3–1.2)	0.76 ± 0.6	0.69(0.16–1.04)	0.54 ± 0.35	0.5(0.19–0.88)	0.7 ± 0.43	0.67(0.33–1.07)	0.821	0.488	-
	**Test Stat./p**	24.105‡	< 0.001*	9.338	< 0.001*	3.404‡	0.182	22.271‡	< 0.001*			
	Effect Size	0.068		0.400		-		0.453				
***BOP***	Baseline	40.18 ± 23.18	27(25–65.34)	48.5 ± 29.98	41.67(19.4–75)	25.06 ± 24.81	12.5(1.47–41.67)	37.09 ± 25.49	33.4(22.9–50)	5.181†	0.159	-
	21 st day	18.9 ± 16.87	15.47(10.42–17.57)	30.82 ± 20.22	29.17(16.67–36.1)	23.74 ± 13.33	25(8.33–33.33)	28.26 ± 27.38	18.7(8.33–50)	4.419†	0.220	-
	28th day	3.08 ± 3.63	0.96(0.17–8.33)	15.82 ± 9.64	14.58(8.33–16.67)	13.07 ± 10.46	8.33(4.17–22.2)	15.54 ± 15.87	12.5(8.33–20.33)	17.604†	< 0.001*	0.101
	**Test Stat./p**	27.559‡	< 0.001*	10.323	< 0.001*	6.140‡	0.050	18.436‡	< 0.001*			
	Effect Size	0.623		0.424		-		0.449				
***PD***	Baseline	2.41 ± 0.75	2.22(1.74–2.82)	2.35 ± 0.74	2.31(1.8–2.64)	2,63 ± 0,75	2,56 (2,32–2,80)	2.64 ± 0.48	2.61(2.33–2.82)	7.561†	0.056	-
	21 st day	1.86 ± 0.45	1.83(1.5–2.17)	2.18 ± 0.98	2.08(1.25–2.44)	1.66 ± 0.53	1.55(1.11–2.25)	2.32 ± 0.55	2.31(1.89–2.61)	9.394†	0.024*	0.143
	28th day	1.62 ± 0.36	1.61(1.25–2.05)	1.96 ± 0.63	1.86(1.67–2.25)	1.47 ± 0.39	1.44(1.11–1.78)	2.18 ± 0.58	2.11(1.86–2.52)	6.048	0.001*	0.388
	**Test Stat./p**	21.217	< 0.001*	10.964‡	0.004*	24.138‡	< 0.001*	14.673	< 0.001*			
	Effect Size	0.602		0.308		0.328		0.512				
***OHIP Scores***	Beginning 7th day 14th day	19.27 ± 13.02 16.73 ± 9.73 7.20 ± 8.65	21(7–32) 21(9–24) 2(0–19)	10.33 ± 8.64 11.33 ± 5.79 3.93 ± 3.28	8(3–19) 10(6–16) 3(1–7)	11.87 ± 8.56 13.47 ± 5.87 12.33 ± 9.18	7(5–21) 14(7–18) 11(5–22)	10.8 ± 5.37 14.53 ± 7.75 5.87 ± 6.29	11(6–14) 16(7–21) 3(2–10)	5.040† 1.361 3.698	0.169 0.264 0.017*	- - 0.165
	**Test Stat./p**	18.542‡	< 0.001*	18.213	< 0.001*	0.195	0.824	15.946	< 0.001*			
	Effect Size	0.439		0.565		-		0.532				

*p < 0.05, †Kruskal–Wallis test and ‡Friedman test, *M* Median. Q: 25th–75th Percentile, *SD* Standard Deviation

Post hoc analyses indicated that GI values were significantly higher at baseline than at later time points in all groups. Similarly, PI and BOP values showed significant reductions over time in the HA, I-PRF, and control groups. PD measurements decreased significantly from baseline to follow-up in all groups, with an additional reduction between days 21 and 28 observed in the HA group.

Regarding OHIP scores, intergroup analyses revealed statistically significant differences among the study groups at day 14 (*p* = 0.017). Post hoc tests showed a significant difference between the I-PRF and T-PRF groups, with higher OHIP scores observed in the I-PRF group (*p* = 0.022).

Time-based analyses regarding OHIP scores revealed statistically significant differences among measurement time points in the HA, I-PRF, and control groups (*p* < 0.05). Post hoc tests showed that, in both the HA and I-PRF groups, day-14 measurements were significantly lower than baseline and day-7 values (*p* ≤ 0.002). In the control group, day-7 values were significantly higher than both baseline and day-14 values, and baseline values were significantly higher than day-14 values (*p* < 0.05).

The distribution of VAS pain and burning scores according to study groups and measurement time points is presented in Table 2. Intergroup analysis revealed statistically significant differences in pain scores on day 6 (*p* = 0.008) and burning scores on day 5 (*p* = 0.008). Post hoc tests showed significant differences between the I-PRF and T-PRF groups for both day-6 pain (*p* = 0.011) and day-5 burning scores (*p* = 0.005), with higher values observed in the T-PRF group.

**Table 2:** Distribution and comparison of VAS pain and burning measurements according to study groups and time points

		Hyaluronic Acid		I-PRF		T-PRF		Control		Between Groups		
VAS	Between Times	Mean ± SD	M.(25%−75%Q.)	Mean ± SD	M.(25%−75%Q.)	Mean ± SD	M.(25%−75%Q.)	Mean ± SD	M.(25%−75%Q.)	Test Statistics	p	Effect Size
Pain	Beginning	3.33 ± 1.35	3(2–4)	4.07 ± 1.83	4(3–6)	4.07 ± 2.4	4(2–6)	2.47 ± 1.3	2(1–4)	6.977	0.073	-
	Day 1	2.87 ± 1.36	2(2–4)	3.27 ± 1.62	3(2–5)	3.73 ± 2.6	3(1–6)	2.4 ± 1.06	3(1–3)	2.383	0.497	-
	Day 2	2.07 ± 1.1	2(1–3)	3.2 ± 1.66	3(2–4)	3.13 ± 2.36	3(1–5)	2.13 ± 1.13	2(1–3)	4.359	0.225	-
	Day 3	1.87 ± 1.3	1(1–3)	2.07 ± 1.16	2(1–3)	3.13 ± 2.72	3(1–4)	1.93 ± 1.58	1(1–2)	2.468	0.481	-
	Day 4	1.47 ± 0.92	1(1–2)	1.6 ± 0.74	1(1–2)	2.67 ± 2.13	2(1–3)	1.33 ± 0.72	1(1–1)	6.946	0.074	-
	Day 5	1.33 ± 0.62	1(1–2)	1.13 ± 0.35	1(1–1)	2.13 ± 1.6	2(1–2)	1.33 ± 0.62	1(1–2)	6.837	0.077	-
	Day 6	1.2 ± 0.77	1(1–1)	1 ± 0	1(1–1)	1.67 ± 0.98	1(1–2)	1.13 ± 0.52	1(1–1)	11.762	0.008*	0.129
	Day 7	1.27 ± 1.03	1(1–1)	1 ± 0	1(1–1)	1.27 ± 0.59	1(1–2)	1 ± 0	1(1–1)	6.568	0.087	-
	Test St./p	61.635	< 0.001*	79.343	< 0.001*	56.629	< 0.001*	42.350	< 0.001*			
	Effect S	0.478		0.663		0.451		0.790				
Burning	Beginning	3.87 ± 1.25	4(3–5)	2.8 ± 0.86	3(2–3)	4.47 ± 2.67	4(2–6)	3.2 ± 1.86	3(1–4)	5.991	0.112	-
	Day 1	3 ± 1.31	3(2–4)	2.67 ± 0.82	3(2–3)	4.47 ± 2.95	4(2–7)	2.33 ± 1.23	2(1–3)	5.039	0.169	-
	Day 2	2.4 ± 1.18	2(2–3)	2.6 ± 1.24	2(2–4)	3.93 ± 3.22	2(1–6)	1.8 ± 0.86	2(1–3)	4.913	0.178	-
	Day 3	1.93 ± 1.62	1(1–2)	1.53 ± 0.52	2(1–2)	3.27 ± 2.69	2(1–5)	1.67 ± 1.4	1(1–2)	6.288	0.098	-
	Day 4	1.8 ± 1.21	1(1–3)	1.13 ± 0.35	1(1–1)	2.73 ± 2.37	2(1–4)	1.6 ± 1.18	1(1–2)	7.339	0.062	-
	Day 5	1.53 ± 1.06	1(1–2)	1 ± 0	1(1–1)	2.33 ± 1.72	2(1–3)	1.4 ± 0.91	1(1–1)	11.738	0.008*	0.170
	Day 6	1.33 ± 0.62	1(1–2)	1 ± 0	1(1–1)	1.67 ± 1.11	1(1–2)	1.33 ± 0.72	1(1–1)	5.831	0.120	-
	Day 7	1.13 ± 0.35	1(1–1)	1 ± 0	1(1–1)	1.33 ± 0.82	1(1–2)	1.27 ± 0.7	1(1–1)	2.514	0.473	-
	Test St./p	68.850	< 0.001*	76.391	< 0.001*	66.057	< 0.001*	42.213	< 0.001*			
	Effect S	0.517		0.657		0.477		0.399				

*p < 0.05 (Data are presented as Mean ± Standard Deviation (SD) and Median (25%−75% Percentiles)

Time-point analysis demonstrated statistically significant differences in both pain and burning scores across measurement days in all groups (*p* < 0.001). Post hoc comparisons revealed that, in the HA, I-PRF, and T-PRF groups, pain and burning scores at later time points, particularly days 5, 6, and 7, were significantly lower compared with baseline and early postoperative measurements (days 1–2) (*p* < 0.001). In the control group, significant differences were mainly observed between day 7 and baseline or day-1 measurements for pain, and between days 5–7 and baseline for burning scores (*p* < 0.001).

The distributions of LTH measurements according to study groups and time points are presented in Table 3. Intergroup analysis revealed statistically significant differences at all measurement time points (day 7: *p* < 0.001; day 14: *p* = 0.003; day 21: *p* = 0.005; day 28: *p* = 0.005).

**Table 3:** Distribution and comparison of LTH measurements according to study groups and time points

	Hyaluronic Acid		I-PRF		T-PRF		Control		Between Groups		
Between Times	Mean ± SD	M.(25%−75%Q.)	Mean ± SD	M.(25%−75%Q.)	Mean ± SD	M.(25%−75%Q.)	Mean ± SD	M.(25%−75%Q.)	Test Statistics	p	Effect Size
Day 7	3.27 ± 0.46	3(3–4)	3.33 ± 0.49	3(3–4)	3.27 ± 0.46	3(3–4)	2.53 ± 0.52	3(2–3)	18.629	< 0.001*	0.333
Day 14	3.73 ± 0.59	4(3–4)	4.07 ± 0.59	4(4–4)	4 ± 0.53	4(4–4)	3.33 ± 0.49	3(3–4)	13.704	0.003*	0.225
Day 21	4.6 ± 0.51	5(4–5)	4.4 ± 0.51	4(4–5)	4.73 ± 0.46	5(4–5)	4.07 ± 0.46	4(4–4)	12.915	0.005*	0.225
Day 28	4.93 ± 0.26	5(5–5)	4.93 ± 0.26	5(5–5)	4.93 ± 0.26	5(5–5)	4.53 ± 0.52	5(4–5)	12.744	0.005*	0.216
Test St./p	39.186	< 0.001*	32.951	< 0.001*	36.744	< 0.001*	38.088	< 0.001*			
Effect Size	0.816		0.714		0.798		0.770				

*p < 0.05 (Data are presented as Mean ± Standard Deviation (SD) and Median (25%−75% Percentiles)

On day 7, the control group (2.53 ± 0.52) exhibited lower LTH values compared with the HA (3.27 ± 0.46), I-PRF (3.33 ± 0.49), and T-PRF (3.27 ± 0.46) groups. On day 14, mean LTH values increased in all groups. The control group (3.33 ± 0.49) remained lower than the HA (3.73 ± 0.59), I-PRF (4.07 ± 0.59), and T-PRF (4.00 ± 0.53) groups. On day 21, LTH measurements further increased, with values of 4.60 ± 0.51 in the HA group, 4.40 ± 0.51 in the I-PRF group, 4.73 ± 0.46 in the T-PRF group, and 4.07 ± 0.46 in the control group. Similarly, on day 28, the control group (4.53 ± 0.52) exhibited lower LTH values than the HA (4.93 ± 0.26), I-PRF (4.93 ± 0.26), and T-PRF (4.93 ± 0.26) groups.

Across time points, all groups showed significant increases in LTH measurements (*p* < 0.001). From day 7 to day 28, mean LTH values increased by 1.66 units in the HA group, 1.60 units in the I-PRF group, 1.66 units in the T-PRF group, and 2.00 units in the control group, indicating progressive healing over time.

The distributions of postoperative non-epithelialized areas across study groups and time points are presented in Table 4. Intergroup analysis revealed statistically significant differences at days 7, 14, 21, and 28 (*p* < 0.05). Post hoc tests showed that on day 7, the control group had significantly larger non-epithelialized areas (169,440 ± 23,583) compared with the HA (122,262 ± 18,363), I-PRF (124,046 ± 28,077), and T-PRF groups (121,525 ± 20,922) (*p* < 0.001). A greater reduction in the non-epithelialized area was observed in the intervention groups; on day 7, the HA group exhibited a 47,178 lower mean value compared with the control group. On day 14, the control group (99,918 ± 13,827) showed significantly higher measurements than I-PRF (56,139 ± 18,206) and T-PRF groups (58,911 ± 13,595) (*p* < 0.001). On day 21, the control group (62,137 ± 13,931) had higher values than the HA (27,902 ± 5500), I-PRF (26,174 ± 8054), and T-PRF groups (29,333 ± 10,972) (*p* < 0.001). On day 28, tests revealed that the control group had significantly higher measurements (16,384 ± 4037) compared with the HA (10,199 ± 1623), I-PRF (8200 ± 2673), and T-PRF groups (8521 ± 2946) (*p* < 0.001).

**Table 4:** Distribution and comparison of wound area measurements according to study groups and measurement times

	Hyaluronic Acid		I-PRF		T-PRF		Control		Between Groups		
Between Times	Mean ± SD	M.(25%−75%Q.)	Mean ± SD	M.(25%−75%Q.)	Mean ± SD	M.(25%−75%Q.)	Mean ± SD	M.(25%−75%Q.)	Test Statistics	p	Effect Size
Beginning	269,998.6 ± 33,175.45	271,990(258,308–279742)	265,708.87 ± 55,144.96	275,995(211,656–318,470)	293,258.27 ± 59,198.47	322,513(231,333–345,506)	262,730.8 ± 38,567.2	243,330(231,313–308,903)	3.639†	0.303	-
Day 7	122,262.4 ± 18,363.03	121,689(109,345–132178)	124,046.27 ± 28,077.39	118,911(106,913–148682)	121,525.4 ± 20,922.33	121,090(110,883–127932)	169,440.8 ± 23,583.35	159,790(150,047–190434)	15.552	< 0.001*	0.454
Day 14	58,470.4 ± 8346.38	58,964(50,218–65412)	56,139 ± 18,206.71	53,284(44,917–67,820)	58,911.53 ± 13,595.14	57,779(53,921–61,882)	99,918.73 ± 13,827.77	96,532(89,519–102,547)	31.115†	< 0.001*	0.647
Day 21	27,902.93 ± 5500.54	27,221(23,482–31,267)	26,174.8 ± 8054.79	24,657(21,947–28,856)	29,333.53 ± 10,972.44	26,547(24,732–28,391)	62,137.73 ± 13,931.97	58,697(55,423–69,515)	31.532†	< 0.001*	0.699
Day 28	10,199.73 ± 1623.27	9835(8776–11,491)	8200.2 ± 2673.93	7834(6178–10,830)	8521.47 ± 2946.83	8034(6822–10,142)	16,384 ± 4037.68	15,677(12,698–20,922)	25.014	< 0.001*	0.573
Test St./p	60.000‡	< 0.001*	60.000‡	< 0.001*	60.000‡	< 0.001*	60.000‡	< 0.001*			
Effect Size	0.983		0.958		0.946		0.975				

*p < 0.05 †Kruskal Wallis test and ‡Friedman test (Data are presented as Mean ± Standard Deviation (SD) and Median (25%−75% Percentiles)

Analysis across time points also demonstrated statistically significant reductions in wound area measurements within all groups over time (*p* < 0.001). The distributions of H₂O₂ foaming test and bleeding status results according to study groups and time points are presented in Table 5. Analysis across groups showed no statistically significant associations between groups and H₂O₂ foaming test results at any time point (*p* > 0.05). Pairwise comparisons are presented in Supplementary Table 3. Analysis across time points revealed statistically significant differences in H₂O₂ foaming test results within all study groups (*p* < 0.05). In the HA group, statistically significant differences were observed between day 7 and day 14 (*p* = 0.031), day 7 and day 21 (*p* < 0.001), and day 14 and day 21 (*p* = 0.032). In the I-PRF group, significant differences were detected between day 7 and day 14 (*p* = 0.032) and between day 7 and day 21 (*p* = 0.012). In the T-PRF group, statistically significant differences were observed between day 7 and day 14 (*p* = 0.048), day 7 and day 21 (*p* < 0.001), and day 7 and day 28 (*p* < 0.001).

**Table 5:** Distribution and associations of bleeding conditions and H_2_O_2_ foaming status according to study groups and measurement time points

		Hyaluronic Acid			I-PRF			T-PRF			Control			Between Groups		
		n	%	%G	n	%	%G	n	%	%G	n	%	%G	Test Statistics	p	Effect size
*Bleeding Conditions*																
*Beginning*	Absent	11a	44.0	73.3	5ab	20.0	33.3	7ab	28.0	46.7	2b	8.0	13.3	11.726**	0.008*	0.442
	Present	4a	11.4	26.7	10ab	28.6	66.7	8ab	22.9	53.3	13b	37.1	86.7			
*Day 1*	Absent	13	27.7	86.7	12	25.5	80.0	12	25.5	80.0	10	21.3	66.7	1.812	0.702	-
	Present	2	15.4	13.3	3	23.1	20.0	3	23.1	20.0	5	38.5	33.3			
*Day 2*	Absent	15	28.8	100.0	12	23.1	80.0	13	25.0	86.7	12	23.1	80.0	3.759	0.340	-
	Present	0	0.0	0.0	3	37.5	20.0	2	25.0	13.3	3	37.5	20.0			
*Day 3*	Absent	15	26.3	100.0	15	26.3	100.0	13	22.8	86.7	14	24.6	93.3	3.047	0.610	-
	Present	0	0.0	0.0	0	0.0	0.0	2	66.7	13.3	1	33.3	6.7			
*Day 4*	Absent	15	26.3	100.0	15	26.3	100.0	13	22.8	86.7	14	24.6	93.3	3.047	0.610	-
	Present	0	0.0	0.0	0	0.0	0.0	2	66.7	13.3	1	33.3	6.7			
*Day 5*	Absent	15	25.9	100.0	15	25.9	100.0	13	22.4	86.7	15	25.9	100.0	3.703	0.233	-
	Present	0	0.0	0.0	0	0.0	0.0	2	100.0	13.3	0	0.0	0.0			
*Day 6*	Absent	15	25.0	100.0	15	25.0	100.0	15	25.0	100.0	15	25.0	100.0	-	-	-
	Present	0	0.0	0.0	0	0.0	0.0	0	0.0	0.0	0	0.0	0.0			
*Day 7*	Absent	15	25.0	100.0	15	25.0	100.0	15	25.0	100.0	15	25.0	100.0	-	-	-
	Present	0	0.0	0.0	0	0.0	0.0	0	0.0	0.0	0	0.0	0.0			
*Between Times*	Test St	22.842			49.143			21.365			62.216					
	p	0.002*			< 0.001*			0.003*			< 0.001*					
	Effect size	0.504			0.739			0.487			0.831					
*H*_*2*_*0*_*2*_* foaming*																
*Day 7*	Absent	1	25.0	6.7	2	50.0	13.3	1	25.0	6.7	0	0.0	0.0	2.134	0.898	-
	Present	14	25.0	93.3	13	23.2	86.7	14	25.0	93.3	15	26.8	100.0			
*Day 14*	Absent	7	25.0	46.7	9	32.1	60.0	8	28.6	53.3	4	14.3	26.7	3.750**	0.290	-
	Present	8	25.0	53.3	6	18.8	40.0	7	21.9	46.7	11	34.4	73.3			
*Day 21*	Absent	14	30.4	93.3	11	23.9	73.3	13	28.3	86.7	8	17.4	53.3	7.179	0.076	
	Present	1	7.1	6.7	4	28.6	26.7	2	14.3	13.3	7	50.0	46.7			
*Day 28*	Absent	15	25.4	100.0	15	25.4	100.0	14	23.7	93.3	15	25.4	100.0	2.855	1.000	-
	Present	0	0.0	0.0	0	0.0	0.0	1	100.0	6.7	0	0.0	0.0			
*Between Times*	Test St		31.531			24.767			28.909			27.792				
	p		< 0.001*			< 0.001*			< 0.001*			< 0.001*				
	Effect Size		0.592			0.525			0.567			0.556				

*p < 0.0, **Pearson Chi-Square test

Group analysis revealed a statistically significant relationship between the study groups and baseline bleeding status (*p* = 0.008). At baseline, the majority of patients in the HA group showed absence of bleeding (73.3%), whereas bleeding was more frequently observed in the control group (86.7%).

Pairwise comparisons of bleeding status over time are presented in Supplementary Table 4. In the I-PRF group, statistically significant differences were observed between baseline and day 1 (*p* = 0.048) and between baseline and day 2 (*p* = 0.048). In the T-PRF group, no statistically significant differences were detected between baseline and the follow-up time points (*p* > 0.05). In the control group, significant differences were observed between baseline and day 2 (*p* = 0.020), day 3 (*p* < 0.001), and day 4 (*p* < 0.001).

Analgesic consumption across study groups and time points is presented in Supplementary Table 5. Intergroup analysis revealed a statistically significant difference on day 1 (*p* = 0.006). Post hoc tests indicated a significant difference between the HA and I-PRF groups (*p* = 0.004), with the HA group exhibiting higher analgesic consumption than the I-PRF group. Analysis across time points revealed significant differences within all study groups (*p* < 0.001). In the HA group, day 1 measurements were higher than days 3–7. In the I-PRF group, day 1 values exceeded those on days 6 and 7. In the T-PRF group, day 1 measurements were higher than days 5–7, and day 2 was higher than day 7. In the control group, day 1 measurements were higher than days 4–7.

## Discussion

Following a surgical gingivectomy, wound healing occurs by secondary intention, which is generally associated with prolonged recovery and increased discomfort compared to the faster and less painful healing process of primary intention [6]. Finding novel, less intrusive treatment techniques that can not only speed up the healing process but also lessen negative effects is an ongoing endeavor, particularly in procedures such as gingivectomy and gingivoplasty, where patient morbidity is a major concern. To the best of our knowledge, no study to date has compared the effects of I-PRF, T-PRF, and HA gel on wound healing following gingivectomy and gingivoplasty.

In this study, wound healing and tissue response were assessed using clinician-reported measures (Mira-2 Tone solution, LTH index, H₂O₂ foaming test, and clinical periodontal parameters) and patient-reported outcomes (pain and burning via VAS, analgesic use, bleeding, and OHIP-14). The use of Mira-2 Tone solution [3, 41], together with the H₂O₂ foaming test [12, 26], allowed visual assessment of epithelialization during the early healing phase, while the LTH wound healing index provided a standardized clinical scoring system for soft-tissue healing [3, 12]. In addition, digital image analysis using ImageJ software enabled objective measurement of non-epithelialized wound areas from standardized clinical photographs [3, 41]. The combination of clinician-reported parameters and patient-reported outcomes (VAS pain/burning scores [12, 44, 45] and OHIP-14 [46]) allowed a comprehensive evaluation of both biological healing and patient-perceived recovery. Similar multimodal approaches have been recommended in previous periodontal wound-healing studies to improve the reliability of clinical outcome assessment [3, 12, 26–28, 40, 41, 43, 47–50]. However, to the best of our knowledge, no previous study has comprehensively evaluated all these clinical, digital, and patient-reported parameters together within the same experimental model.

Gingivectomy procedures create a standardized surgical wound that heals by secondary intention, making them a suitable clinical model for evaluating the effects of different biologic agents on soft tissue healing [6]. Wound healing requires essential processes such as cell differentiation, proliferation, migration, and collagen deposition. Meanwhile, HA, a member of the glycosaminoglycans (GAGs) family and a key component of the extracellular matrix (ECM), supports this process by reducing inflammation, enhancing vascularization, and promoting collagen synthesis [51]. Alpan et al. reported that high molecular weight 0.6% HA accelerated wound healing more than hypochlorous acid spray and flurbiprofen spray in their study evaluating palatal wound healing after free gingival graft (FGG) surgery [47]. Yıldırım et al. demonstrated that the topical application of both 0.2% and 0.8% HA significantly reduces postoperative pain and burning sensation while promoting palatal wound healing following free FGG procedures [43]. Hassan et al. reported that PRF gave better results than HA in terms of pain reduction and healing properties after FGG [52]. Similarly, Soliman et al. reported that PRF was superior to 0.2% HA in terms of wound healing in the early period (1st week) after FGG [50]. When studies evaluating the efficacy of HA after gingivectomy are reviewed, Yakout et al. reported that the adjunctive application of 2% HA gel to photobiomodulation therapy resulted in significantly improved wound healing after gingivectomy compared with photobiomodulation therapy alone [6]. Kale et al. revealed that topical application of PRF and HA gel to the wound area after depigmentation resulted in faster healing and better patient comfort compared to the use of periodontal dressing alone [49]. Our study also supports the existing literature by demonstrating that the use of HA positively contributes to periodontal wound healing. In the present study, the HA group exhibited significant time-dependent improvements in key clinical parameters, including gingival index, plaque index, bleeding on probing, soft tissue color difference, epithelialization, and patient-reported outcomes. Compared with the control group, HA application resulted in reduced non-epithelialized wound areas, lower bleeding tendency, improved tissue healing dynamics, and favorable pain and burning score trends over the postoperative period. Although platelet-derived concentrates showed superior outcomes in certain parameters, the overall findings confirm that topical HA is an effective adjunct in enhancing postoperative wound healing and patient comfort, in line with previous clinical evidence.

In this study, I-PRF and T-PRF were selected as autologous platelet concentrates that provide growth factors and cytokines directly to the wound site, supporting angiogenesis and epithelialization, and allowing clinical evaluation of their effects on soft tissue healing. Clinical evidence regarding the use of I-PRF in periodontal soft tissue wound healing remains relatively scarce; however, a recent randomized controlled clinical study reported that adjunctive I-PRF application significantly improved epithelial wound healing after gingivectomy and gingivoplasty, as demonstrated by superior clinical healing indices and reduced wound staining compared with surgery alone [3]. Beyond injectable formulations, other autologous platelet concentrates have also been explored for their potential role in soft tissue healing. In this context, T-PRF has attracted growing interest. Although the number of studies evaluating T-PRF in periodontal and oral surgical applications, particularly with respect to wound healing, remains limited, the available data indicate encouraging regenerative and wound-healing outcomes. Ustaoglu et al. reported that T-PRF applied to the palate after FGG significantly accelerated mucosal tissue healing [28]. Recent studies have reported that T-PRF applied with a coronally positioned flap in the treatment of gingival recessions may be an alternative to subepithelial connective tissue graft and may accelerate wound healing [29, 53]. Ünsal et al. demonstrated by ultrasonography that T-PRF applied to the palate after FGG harvesting increased tissue thickness and vascularisation density [30]. Ustaoğlu et al. reported that T-PRF applied to extraction sockets accelerates early wound healing and can be used in alveolar socket preservation [40]. Arabacı et al. reported that T-PRF applied in open flap debridement (OFD) increased the growth factors and decreased the RANKL/OPG ratio in GCF [54]. Similarly, Eminoğlu et al. reported that the combination of T-PRF with the OFD was more successful in terms of clinical, radiographic, and biochemical measurements of periodontal regeneration compared to the procedure alone [55]. In the present study, T-PRF application resulted in more favorable wound healing outcomes compared to the control group, particularly in terms of reduced non-epithelialized areas, lower pain and burning sensation scores, and improved LTH healing indices over time. Although some early postoperative discomfort parameters were higher in the T-PRF group compared to I-PRF, these differences diminished during later healing phases, suggesting a time-dependent positive effect of T-PRF on tissue maturation. Overall, the findings of this study support the adjunctive use of T-PRF as a biocompatible and effective biomaterial for enhancing soft-tissue wound healing, in line with the limited but promising evidence reported in previous periodontal and oral surgical studies.

When the studies comparing different treatment modalities are examined, it becomes evident that the reported outcomes are heterogeneous and vary considerably depending on the applied approach. Şen et al. demonstrated that adjunctive application of HA or I-PRF at palatal donor sites significantly reduced postoperative morbidity and enhanced wound healing compared to controls, with HA showing superior clinical outcomes, while I-PRF also effectively reduced postoperative pain [12]. Chatterjee et al. showed that PRF and T-PRF applied in flap operation in the treatment of intra-osseous defects showed similar clinical and radiological results and showed a significant improvement compared to the control group [56]. Similarly, Mitra et al. reported that both T-PRF and PRF applied with OFD showed positive clinical and radiological results in their split-mouth designed study, but there was no significant difference between the two [57]. In line with these heterogeneous findings, the results of the present study further support the notion that platelet-derived biomaterials exert beneficial effects on postoperative healing, although their clinical performance may differ depending on the formulation and application method. In the current study, the T-PRF group demonstrated more favorable outcomes than the control group across several wound-healing–related parameters, including reduced non-epithelialized areas and improved healing indices over time, while also showing a comparable clinical course to other adjunctive modalities. These findings are consistent with previous reports indicating that T-PRF provides meaningful improvements in soft tissue healing without necessarily exhibiting clear superiority over other platelet concentrates, thereby underscoring its potential as a reliable and biologically sound adjunct in periodontal wound management.

The present study has several limitations that should be acknowledged when interpreting the results. First, several outcome measures, including VAS pain and burning scores as well as OHIP assessments, are inherently subjective and may not fully reflect objective wound healing dynamics. Although statistically significant differences were observed among the groups, some of these differences may have limited clinical relevance. In addition, the use of locally applied therapeutic agents in the intervention groups may have contributed to lower patient-reported discomfort scores compared to the control group. Consequently, the assessment of wound healing and epithelialization relied on clinical indices such as the LTH index and Mira-2 tone staining, which, while clinically validated, lack direct histological correlation at the cellular level. In addition, biochemical or immunological analyses (e.g., cytokine levels or collagen deposition) were not included in the study design; therefore, the underlying biological mechanisms associated with the observed clinical outcomes could not be directly explored. Furthermore, while the 28-day follow-up period is a well-established timeframe in recent literature for evaluating early wound healing and acute patient-reported outcomes [2, 3, 26], it may not fully capture the long-term maturation and definitive stability of the gingival margin. The short follow-up period may further restrict the generalizability of the findings and the assessment of long-term healing outcomes. Although photographic documentation was standardized by using the same camera, angle, distance, and lighting conditions, the possibility of minor human-related errors cannot be completely excluded. In addition, no customized stent was used to standardize the clinical measurements, which may have introduced variability in the assessment of clinical parameters across different time points. Finally, despite efforts to standardize surgical procedures, complete uniformity in wound characteristics could not be fully achieved, which may have influenced healing responses.

### Clinical relevance

From a clinical perspective, the findings of the present study indicate that adjunctive use of biologically active materials such as HA and platelet-derived concentrates (I-PRF and T-PRF) may enhance soft-tissue healing and improve patient comfort following gingivectomy procedures. Although all tested modalities demonstrated beneficial effects on wound healing compared with the control group, important practical considerations may influence their clinical selection. HA can be applied easily without the need for blood collection, centrifugation, or additional chairside preparation, which may make it a more convenient option in routine clinical practice. However, as a commercially available product, its use may be associated with additional cost depending on the formulation and healthcare setting. In contrast, platelet-derived biomaterials such as I-PRF and T-PRF require venous blood collection and centrifugation but are autologous and biologically active materials that provide growth factors directly to the surgical site. These procedures may involve additional equipment, time, and consumable costs, such as centrifuge tubes and sterilization requirements. Therefore, while the present results support the beneficial role of all three modalities in enhancing postoperative healing, the choice of adjunctive treatment may depend on clinical logistics, practitioner preference, and cost–benefit considerations in daily periodontal practice.

## Conclusion

Based on the observed differences in wound healing and patient-reported outcomes, the null hypothesis was rejected. Within the study’s limitations, HA, I-PRF, and T-PRF all positively influenced gingival wound healing after gingivectomy and gingivoplasty. HA showed consistent clinical and patient-reported benefits, while platelet-derived biomaterials, particularly T-PRF, promoted wound maturation and epithelialization. Differences among these modalities highlight the impact of formulation and application protocols. Further large-scale, long-term studies are needed to clarify their comparative effectiveness.

## Supplementary Information

Below is the link to the electronic supplementary material.

Supplementary file1 (DOCX 20 KB)

Supplementary file2 (DOCX 19 KB)

Supplementary file3 (DOCX 19 KB)

Supplementary file4 (DOCX 16 KB)

## Data Availability

The full trial protocol relevant to the study is available from the corresponding author upon request.
