# Correction to: Comparison of injectable platelet-rich fibrin, titanium platelet-rich fibrin, and 0.8% hyaluronic acid applications versus periodontal dressing alone in wound healing after gingivectomy and gingivoplasty operations: randomized controlled clinical study

**DOI:** 10.1007/s00784-026-06887-8

**Published:** 2026-04-23

**Authors:** Özlem Saraç Atagün, Şeyma Çardakci Bahar, Seval Ceylan Şen, Gülbahar Ustaoğlu, Erkan Özcan, Zeynep Cansu Güzel, Merve Inceöz

**Affiliations:** Department of Periodontology, Gülhane Faculty of Dentistry, University of Health Sciences, Ankara, Turkey

**Correction to: Clin Oral Invest**

10.1007/s00784-026-06860-5

In the published paper, an error occurred in Figure 4 of the original article. Due to a mix-up of the photographs during the assembly of the final figure, the images were incorrectly placed. This does not affect the results, discussions, or the scientific conclusions of the study. The authors apologize for this error.

The original article has been corrected.

