# Supplementary material for: Comparison of injectable platelet-rich fibrin, titanium platelet-rich fibrin, and 0.8% hyaluronic acid applications versus periodontal dressing alone in wound healing after gingivectomy and gingivoplasty operations: randomized controlled clinical study
Source: Clin Oral Investig. 2026 Apr 14;30(5):174. doi: 10.1007/s00784-026-06860-5 (PMC13079500; doi:10.1007/s00784-026-06860-5)
Supplement: Supplementary file 1 — Supplementary file1 (DOCX 20 KB) [file 784_2026_6860_MOESM1_ESM.docx]

**Supplementary Table 1:** Distribution and comparison of ages by groups

|  |  | Mean±S.D. | Test Statistics | p |
| --- | --- | --- | --- | --- |
| HA |  | 23.13±5.68 | 1.011 | 0.799 |
| I-PRF |  | 22.27±7.44 |  |  |
| T-PRF |  | 22.93±7.83 |  |  |
| Control |  | 21.27±5.54 |  |  |

The age distributions of the study groups are presented in the table. and comparisons were performed using the Mann–Whitney U test. The analysis revealed no statistically significant difference in age between the study groups (p > 0.05).

**Supplementary Table 2:** Gender distribution and relationships between genders according to groups

|  | Female | | | Male | | |  |  |
| --- | --- | --- | --- | --- | --- | --- | --- | --- |
|  | n | % | %C. | n | % | %C. | Test Statistics | p |
| HA | 8 | 53.3 | 25.8 | 7 | 46.7 | 24.1 | 0.200 | 0.978 |
| I-PRF | 8 | 53.3 | 25.8 | 7 | 46.7 | 24.1 |  |  |
| T-PRF | 8 | 53.3 | 25.8 | 7 | 46.7 | 24.1 |  |  |
| Control | 7 | 46.7 | 22.6 | 8 | 53.3 | 27.6 |  |  |

% = Row percentage, %C = Column percentage

The distribution of genders according to work groups is provided in the table, and the Pearson Chi-Square test was applied to examine the relationships between them. The analyses revealed no statistically significant relationships between work groups and gender (p>0.05).
