# Supplementary material for: Comparison of injectable platelet-rich fibrin, titanium platelet-rich fibrin, and 0.8% hyaluronic acid applications versus periodontal dressing alone in wound healing after gingivectomy and gingivoplasty operations: randomized controlled clinical study
Source: Clin Oral Investig. 2026 Apr 14;30(5):174. doi: 10.1007/s00784-026-06860-5 (PMC13079500; doi:10.1007/s00784-026-06860-5)
Supplement: Supplementary file 2 — Supplementary file2 (DOCX 19 KB) [file 784_2026_6860_MOESM2_ESM.docx]

**Supplementary Table 5:** Distribution and comparison of analgesic intake according to groups and measurement times

|  | Hyaluronic Acid | | I-PRF | | T-PRF | | Control | | Between Groups | |  |
| --- | --- | --- | --- | --- | --- | --- | --- | --- | --- | --- | --- |
| Between Times | Mean ± SD | M.(25%-75%Q.) | Mean ± SD. | M.(25%-75%Q.) | Mean ± SD | M.(25%-75%Q.) | Mean ± SD | M.(25%-75%Q.) | Test Statistics | p | Effect Size |
| Day 1 | 1.60±0.74 | 2(1-2) | 0.60±0.74 | 0(0-1) | 1.33±0.9 | 1(1-2) | 1±0.65 | 1(1-1) | 12.368 | 0.006* | 0.205 |
| Day 2 | 0.93±0.80 | 1(0-2) | 0.47±0.74 | 0(0-1) | 1.07±1.22 | 1(0-2) | 0.4±0.51 | 0(0-1) | 5.682 | 0.128 | - |
| Day 3 | 0.60±0.83 | 0(0-1) | 0.27±0.59 | 0(0-0) | 0.73±1.1 | 0(0-1) | 0.4±0.51 | 0(0-1) | 2.565 | 0.464 | - |
| Day 4 | 0.47±0.74 | 0(0-1) | 0.27±0.59 | 0(0-0) | 0.53±0.83 | 0(0-1) | 0.07±0.26 | 0(0-0) | 5.153 | 0.161 | - |
| Day 5 | 0.40±0.74 | 0(0-1) | 0.27±0.59 | 0(0-0) | 0.4±0.83 | 0(0-1) | 0±0 | 0(0-0) | 4.693 | 0.196 | - |
| Day 6 | 0.27±0.70 | 0(0-0) | 0.07±0.26 | 0(0-0) | 0.2±0.56 | 0(0-0) | 0±0 | 0(0-0) | 2.448 | 0.485 | - |
| Day 7 | 0.13±0.35 | 0(0-0) | 0±0 | 0(0-0) | 0±0 | 0(0-0) | 0±0 | 0(0-0) | 6.103 | 0.107 | - |
| Test St./p | 57.737 | <0.001* | 22.830 | <0.001* | 52.330 | <0.001* | 50.118 | <0.001* |  |  |  |
| Effect Size | 0.632 |  | 0.250 |  | 0.487 |  | 0.556 |  |  |  |  |

*p<0.05
