# Supplementary material for: Comparison of injectable platelet-rich fibrin, titanium platelet-rich fibrin, and 0.8% hyaluronic acid applications versus periodontal dressing alone in wound healing after gingivectomy and gingivoplasty operations: randomized controlled clinical study
Source: Clin Oral Investig. 2026 Apr 14;30(5):174. doi: 10.1007/s00784-026-06860-5 (PMC13079500; doi:10.1007/s00784-026-06860-5)
Supplement: Supplementary file 3 — Supplementary file3 (DOCX 19 KB) [file 784_2026_6860_MOESM3_ESM.docx]

**Supplementary Table 4:** Holm–Bonferroni corrected McNemar test results

|  | Hyaluronic Acid | I-PRF | T-PRF | Control |
| --- | --- | --- | --- | --- |
|  | p | p | p | p |
| Beginning- Day 1 | 0.500 | 0.048* | 0.536 | 0.080 |
| Beginning- Day 2 | - | 0.048* | 0.300 | 0.020* |
| Beginning- Day 3 | - | - | 0.120 | <0.001* |
| Beginning- Day 4 | - | - | 0.300 | <0.001* |
| Beginning- Day 5 | - | - | 0.467 | - |
| Beginning- Day 6 | - | - | - | - |
| Beginning- Day 7 | - | - | - | - |
| Day 1- Day 2 | - | 1.000 | 1.000 | 0.500 |
| Day 1- Day 3 | - | - | 1.000 | 0.250 |
| Day 1- Day 4 | - | - | 1.000 | 0.250 |
| Day 1- Day 5 | - | - | 1.000 | - |
| Day 1- Day 6 | - | - | - | - |
| Day 1- Day 7 | - | - | - | - |
| Day 2- Day 3 | - | - | 1.000 | 0.500 |
| Day 2- Day 4 | - | - | 1.000 | 0.500 |
| Day 2- Day 5 | - | - | 1.000 | - |
| Day 2- Day 6 | - | - | - | - |
| Day 2- Day 7 | - | - | - | - |
| Day 3- Day 4 | - | - | - | 1.000 |
| Day 3- Day 5 | - | - | 1.000 | - |
| Day 3- Day 6 | - | - | - | - |
| Day 3- Day 7 | - | - | - | - |
| Day 4- Day 5 | - | - | 1.000 | - |
| Day 4- Day 6 | - | - | - | - |
| Day 4- Day 7 | - | - | - | - |
| Day 5- Day 6 | - | - | - | - |
| Day 5- Day 7 | - | - | - | - |
| Day 6- Day 7 | - | - | - | - |

*p<0.05
