# Supplementary material for: Comparison of injectable platelet-rich fibrin, titanium platelet-rich fibrin, and 0.8% hyaluronic acid applications versus periodontal dressing alone in wound healing after gingivectomy and gingivoplasty operations: randomized controlled clinical study
Source: Clin Oral Investig. 2026 Apr 14;30(5):174. doi: 10.1007/s00784-026-06860-5 (PMC13079500; doi:10.1007/s00784-026-06860-5)
Supplement: Supplementary file 4 — Supplementary file4 (DOCX 16 KB) [file 784_2026_6860_MOESM4_ESM.docx]

**Supplementary Table 4:** Holm–Bonferroni corrected McNemar test results

|  | Hyaluronic Acid | I-PRF | T-PRF | Control |
| --- | --- | --- | --- | --- |
|  | p | p | p | p |
| Day 7- Day 14 | 0.031* | 0.032* | 0.048* | - |
| Day 7- Day 21 | <0.001* | 0.012* | <0.001* | - |
| Day 7- Day 28 | - | - | <0.001* | - |
| Day 14- Day 21 | 0.032* | 0.625 | 0.126 | 0.289 |
| Day 14- Day 28 | - | - | - | - |
| Day 21- Day 28 | - | - | 1.000 | - |

*p<0.05
